# Dynamic Weathering Behavior of Heat-Treated Chinese Fir: Surface Properties, Chemical Composition, and Microstructure

**DOI:** 10.3390/polym17233143

**Published:** 2025-11-26

**Authors:** Yun Liu, Chenggong Gao, Qingbo Wang, Bekbolati Hadili, Yufeng Miao, Xinjie Cui, Junji Matsumura

**Affiliations:** 1 Key Laboratory of Wooden Materials Science and Engineering of Jilin Province, College of Material Science and Engineering, Beihua University, Jilin 132013, China; liuyun@beihua.edu.cn (Y.L.); chenggong_gao@163.com (C.G.); 17699716970@163.com (B.H.); 13208623393@163.com (Y.M.); 2 Key Laboratory of Bio-Based Material Science & Technology (Ministry of Education), College of Materials Science and Engineering, Northeast Forestry University, Harbin 150040, China; 3 Shandong Institute for Product Quality Inspection, Jinan 250102, China; 13808931944@163.com; 4 Laboratory of Wood Science, Faculty of Agriculture, Kyushu University, 744 Motooka, Nishi-ku, Fukuoka 819-0395, Japan; matumura@agr.kyushu-u.ac.jp

**Keywords:** artificial weathering, heat treatment, color, gloss, chemistry, structure

## Abstract

Heat-treated wood is widely used for its eco-friendliness and aesthetic appeal. However, it is prone to fading under ultraviolet (UV) radiation, which diminishes its decorative effect and shortens its service life. To clarify the performance evolution and degradation mechanisms of heat-treated wood, Chinese fir, treated at 190 °C under vacuum, was subjected to artificial weathering, and the dynamic changes in surface appearance, chemical composition, and microstructure were monitored. The results show that after artificial weathering, both heat-treated and untreated wood exhibited similar surface color change trends, characterized by darkening, yellowing, and reddening, but heat treatment significantly inhibited surface color changes. After 372 h of weathering, the total color change of heat-treated wood (ΔE = 24.05) was smaller than that of the untreated wood (ΔE = 37.78), and its yellowness index (b* = 58.93%) was also far lower than the untreated group (b* = 119.85%). Additionally, the gloss of heat-treated Chinese fir increased by 17% during weathering. However, as weathering progressed, the protective effect of heat treatment on the appearance gradually weakened, and it could not prevent long-term structural breakdown. The improved color stability is closely linked to condensed lignin and unsaturated phenolic chromophores, while the preferential thermal decomposition of hemicelluloses creates “weathering-vulnerable zones”. This study provides both a theoretical basis for understanding the weathering behavior of heat-treated wood and practical guidance for optimizing wood protection technologies.

## 1. Introduction

The use of wood can lessen the environmental and climate impacts of the construction industry. Compared with other building materials, it offers a high strength-to-weight ratio, natural aesthetic value, and outstanding processing properties, while also being environmentally friendly, renewable, and recyclable. As sustainable development becomes a global consensus, the advantages of wood are more prominent than ever.

However, unprotected wood is susceptible to a wide range of weathering factors, including solar radiation (e.g., ultraviolet, visible, and infrared light), moisture fluctuations (e.g., rain, snow, and frost), temperature variations, biological action, and pollutant emissions. The combination of these factors can lead to wood surface color changes, roughening, cracking, and even degradation of mechanical properties and structural damage. Among them, the ultraviolet (UV) of solar radiation has a particularly significant effect on the surface of wood [1]. UV has high energy and can penetrate to a depth of about 75 µm on the wood surface, triggering photochemical reactions. Due to its aromatic structure, lignin is the main UV-absorbing component of wood, accounting for 80–95% of the wood’s light absorption [2]. It is also the main substance responsible for wood discoloration. Muller et al. [3] pointed out that there is a high correlation between photo-yellowing and lignin degradation in wood. They also noted that carbonyl absorption bands can be formed up to a depth of 120 µm, which exceeds the penetration depth of UV in wood. Quinones formed during lignin degradation [4] further absorb UV and thus promote photo-yellowing of wood. Although most of the UV is absorbed by lignin, a small portion is absorbed by polysaccharides (5–12%) and extractives (2%) in the wood. It is worth noting that aromatic compounds contained in the extracts, such as flavonoids, tannins, astragalus, lignans, quinones, and simple phenols [5], not only absorb UV rays but also scavenge free radicals [6,7,8,9]. Therefore, the extracts, which contain these compounds, are often used as UV absorbers. In addition, the degradation and oxidation of polysaccharides can further exacerbate the deterioration of the wood surface [10], which ultimately leads to a shorter service life of the wood.

Traditional chemical modification methods involve introducing chemical reagents into wood, substances that may gradually leach out during service. Therefore, it is essential to consider their potential impact on the ecological environment and subsequent waste recycling issues. In contrast, heat treatment is an environmentally friendly method of wood modification, which is usually carried out in an oxygen-deficient environment (e.g., noble gas, vacuum, water vapor, water, or vegetable oil) at temperatures ranging from 160 °C to 240 °C. The properties of thermowood are closely related to heat-treatment parameters. Studies have shown that temperature and time are the most critical factors in determining the effectiveness of heat treatment compared to other heat treatment parameters, with the effect of temperature being more pronounced than that of treatment time [11]. This conclusion was also pointed out in previous studies; wood properties such as mass loss [12], mechanical properties [13], and hygroscopicity [14] were more significantly affected by heat treatment temperature than by treatment time. This dominant role of temperature stems from the complex chemical reactions of the wood components during the heat treatment process. During heat treatment, hemicellulose, the main component of wood, is most susceptible and is the first to undergo degradation [15], generating carbonyl and carboxyl groups, which are one of the most important causes of wood discoloration [16]. The removal of acetyl groups from hemicellulose was accompanied by the production of acetic acid, which further promoted cellulose degradation. The degradation of hemicellulose and cellulose resulted in a decrease in the number of hydroxyl groups in the cell wall, which significantly diminished the hygroscopicity of the wood and enhanced its dimensional stability. In addition, color changes in thermowood have been associated with lignin condensation, demethoxylation, and changes in certain extractives [17]. Degradation of wood chemistry components reduces food sources for insects and microorganisms, while reduced hygroscopicity limits microbial growth, thereby enhancing the biological durability of wood to some extent [18,19]. Most importantly, heat treatment not only enhances the properties of the wood but also gives it an attractive dark color, making thermowood popular for outdoor construction applications such as decking, railings, and exterior cladding.

With the expanding range of applications of thermowood, its performance in use has attracted much attention. Studies have shown that heat treatment can significantly improve the surface quality of wood [20], especially in improving color stability. Studies [21,22,23] have shown that heat-treatment wood generally exhibits superior color stability under accelerated weathering compared to untreated wood. Mastouri et al. [24] also reported that heat treatment can effectively postpone color changes in wood induced by light radiation, thereby preserving its aesthetic properties in the short term.

However, Yildiz et al. [25] and Kamperidou et al. [26] pointed out that although heat treatment can retard the surface deterioration of wood during weathering, its effectiveness against UV aging and moisture erosion is limited. This contradictory phenomenon indicates that, though heat treatment improves the initial properties of wood, it is difficult to maintain stable surface capability under continuous weathering. Based on this, an in-depth analysis of the performance evolution and degradation mechanism of heat-treated wood during weathering not only contributes theoretically to the wood weathering evaluation system but also provides a foundation for developing new protection methods. Despite significant progress, most studies have focused on comparing the properties of heat-treated wood before weathering and at the “endpoint” (e.g., final color values). However, systematic and continuous tracking of how surface characteristics (such as color and gloss) dynamically evolve during weathering, as well as investigation into the intrinsic relationship between this evolution and microstructural/chemical changes, remains lacking.

Given the uncontrollable and non-reproducible nature of natural climatic conditions, this study conducted an accelerated weathering test using an ultraviolet (UV) light weathering chamber in accordance with ASTM G154 [27]. Dynamic performance evolution and degradation mechanisms of heat-treated wood were analyzed by evaluating color changes (L*, a*, b*, ΔE), surface gloss attenuation, chemical composition evolution, and microstructural degradation of thermowood.

## 2. Materials and Methods

Chinese fir (*Cunninghamia lanceolata* (Lamb.) Hook.) sapwood was selected as the experimental material. The Chinese fir was sourced from plantation forests in the Guiyang area of Guizhou Province, China, and is approximately 25 years old. It has been air-dried (moisture content 8–12%). Samples were collected from the tree trunk at a height of 1.5 m above the ground. Specimens with uniform color, straight grain, and no significant defects were selected for the following experiments. The experiment was conducted at the East Campus of Beihua University, Jilin City, Jilin Province (43.83° N, 126.57° E). The wood was sawn to the required dimensions, and its surface was sanded with 240-grit sandpaper to achieve a smooth finish. Before testing, the samples were placed in a drying oven and treated at 60 °C until they reached an oven-dry state, with the drying time depending on the size of the samples. The samples were divided into two groups: a heat-treated wood group referred to later as TW, and an unheated wood group (unheated-treated wood), which served as the control group and was referred to as UW.

### 2.1. Vacuum Heat Treatment

The samples were heat-treated using a vacuum drying oven (DZF-6020BE, Shanghai Kuntian Laboratory Instrument Co., Ltd., Shanghai, China). The specific steps were as follows: the samples were placed into the vacuum drying oven, which was then subjected to vacuum treatment at a negative pressure of −0.1 MPa. Then, the temperature was raised to 190 °C at a heating rate of approximately 1.3 °C/min, followed by a constant temperature treatment for 2 h (190 °C is the optimal temperature for the heat treatment of Chinese fir [28], at which the mass loss is relatively low, and the basic strength and service performance of the wood can be ensured). After reaching the predetermined time, the power supply of the vacuum drying oven was turned off and allowed to cool down to room temperature naturally. To prevent oxidation and combustion of the wood at high temperatures, the entire treatment process must be maintained under vacuum conditions. These samples were used for subsequent experiments.

### 2.2. Artificially Accelerated Weathering

Artificially accelerated weathering tests were conducted on the samples using a UV accelerated weathering chamber (ASR-2134A, Guangdong Asirui Instrument Technology Co., Ltd., Guangdong, China). Each cycle consisted of two stages: the first stage involved UV irradiation for 8 h (UVA-340, blackboard temperature 60 ± 2 °C, light intensity 0.76 W/m^2^); the second stage was a constant temperature condensation for 4 h (blackboard temperature 50 ± 2 °C). The total duration of artificial weathering was 372 h. After artificial weathering, the samples were placed in a drying oven and treated until they reached an oven-dry state and then cooled to room temperature (25 ± 2 °C) in a desiccator for subsequent testing.

### 2.3. Color Measurement

According to the CIE Lab system [29], color measurements (L*, a*, b*) were taken on the tangential surface of the samples using a chroma meter (SR-62, Shenzhen Sanen Technology Co., Ltd., Shenzhen, China) with a measurement geometry of d/8° integrating sphere. The measurement aperture was positioned perpendicular to the tangential surface of the samples. The dimensions of the samples were 15 cm (longitudinal) × 1.5 cm (radial) × 3.5 cm (tangential). Five replicate samples per group, each divided equally into five regions, and each region was measured ten times and averaged. The samples were measured after 0, 24, 48, 96, 144, 180, 228, 276, 324, and 372 h of weathering. The total color change (ΔE) is calculated using Equation (1):

(1) $$\Delta E=\sqrt{{\left(\Delta L*\right)}^{2}+{\left(\Delta a*\right)}^{2}+{\left(\Delta b*\right)}^{2}}$$

where L* is lightness (0 is black, 100 is white), a* is red-green chromaticity (a + is an increase in redness, a—is an increase in greenness), and b* is yellow-blue chromaticity (b + is an increase in yellowness, b—is an increase in blueness).

### 2.4. Gloss Measurement

The gloss of the tangential surfaces of the samples was measured using a portable glossmeter (KGZ-1B, Tianjin KQ High-tech Co., Ltd., Tianjin, China) at a measurement angle of 60°. The dimensions and quantity of the samples were consistent with those described in Section 2.3. The glossmeter was calibrated using a standard board before measurement, and the average value was taken after 20 measurements.

### 2.5. Determination of Ultraviolet–Visible Absorption Spectroscopy (UV-Vis) of Water-Soluble Extracts of Samples

The extraction procedure for water-soluble components was as follows: the samples were placed in deionized water at a ratio of 1:200 (*m*/*v*) and then subjected to extraction by standing in the dark at 25 ± 2 °C for 48 h. The extract was filtered using a microporous membrane with a pore size of 0.22 μm (which had been rinsed with deionized water before use) to obtain the water-soluble extract. A UV-Vis spectrophotometer (UV-2600i, SHIMADZU, Kyoto, Japan) (software: LabSolutions UV-Vis) was used to scan the water-soluble extract in the wavelength range of 200–600 nm (descending order, medium scanning speed). Deionized water was used as the reference to measure the water-soluble extracts of samples that had been subjected to artificial weathering for 0, 24, 96, 180, and 372 h, with two replicate determinations for each time point.

### 2.6. Attenuated Total Reflectance Fourier Transform Infrared Spectroscopy (ATR-FTIR)

The chemical composition of the samples was determined using a Fourier Transform Infrared Spectrometer (IRAffinity-1S, SHIMADZU, Kyoto, Japan) (software: LabSolutions IR) equipped with a single-crystal diamond accessory (Quest ATR, SPECAC, Orpington, Kent, UK). The measurements were performed over the range of 4000 to 400 cm^−1^ with a resolution of 4 cm^−1^ and 32 scans. Samples subjected to artificial weathering for 0, 24, 96, 180, and 372 h were analyzed, with nine replicate measurements taken at each time point. The infrared spectra were processed using OMNIC software (OMNIC 9.2, Thermo Nicolet, Madison, WI, USA).

### 2.7. X-Ray Diffractometer (XRD)

The crystallinity of the samples was determined using an X-ray diffractometer (X’Pert Pro, PANalytical, Almelo, OV, Netherlands) with an anode Cu target. The measurements were performed using Cu Kα radiation (λ = 0.15418 nm). The scanning parameters included a range of 5° to 90°, a speed of 3.6°/min, and an X-ray tube voltage and current setting of 40 kV and 40 mA. The dimensions of the samples were 1 cm (longitudinal) × 2 mm (radial) × 1 cm (tangential). Specimens that underwent artificial weathering for durations of 0, 24, 96, 180, and 372 h were examined, with three replicate measurements taken at each time point.

### 2.8. Scanning Electron Microscope (SEM)

The cross-section, radial section, and tangential sections of the samples with dimensions of 1 cm (longitudinal) × 1 cm (radial) × 1 cm (tangential) were smoothed using a sliding microtome. The surface morphology of the three sections of the samples during the weathering process was observed using a Scanning Electron Microscope (SEM) (ZEISS Sigma 360 VP, Carl Zeiss AG, Oberkochen, BW, DE). Due to the poor conductivity of the samples, their surfaces were coated with platinum (Pt) to enhance conductivity. To ensure the required vacuum conditions of the instrument, the sample chamber was evacuated to a high vacuum state. The three sections of samples subjected to artificial weathering for 0, 24, 96, 180, and 372 h were analyzed.

## 3. Results and Discussion

### 3.1. Surface Color and Gloss Changes

**Figure 1:**
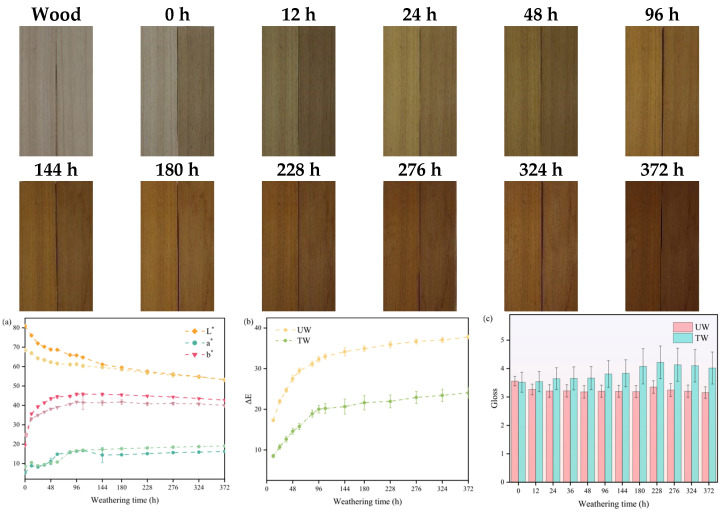
Both groups of samples at different weathering times: photographs (left: UW, right: TW), and quantified (**a**) L*, a*, b*, (**b**) ΔE (UW is represented by – –; TW is represented by –·–), (**c**) gloss.

In the evaluation of material color, the color difference ΔE ≥ 5 is considered a significant color change. As shown in Table 1, the ΔE value of the wood after heat treatment reached 14.27, indicating a significant change in wood color. After heat treatment, the lightness (L*) decreased by 15.59%, while the red-green chromaticity (a*) and yellow-blue chromaticity (b*) increased by 68.41% and 28.62%, respectively. These changes collectively resulted in the wood’s color shifting from light brown to grayish-brown, as shown in the digital photograph for 0 h in Figure 1. These color changes originated from the degradation and oxidation of wood chemical components during the heat treatment process, with the alterations in polysaccharides having the most pronounced impact on color [30]. Regarding the decline in lightness, Sundqvist et al. [31] noted that organic acids such as acetic and formic acid generated during heat treatment can cause the wood to darken, with the degree of darkening being related to the concentration of these acids. Additionally, research by Kačíková et al. [32] further confirmed that the formation of hemicellulose degradation products, lignin condensation, oxidation of phenolic hydroxyl groups (-OH) (such as the formation of quinone compounds), and changes in extractives all contribute to the tendency of wood to darken. In addition to color changes, the gloss of the samples also underwent significant changes. After heat treatment, the gloss of the wood decreased (by 15.08%), a result that is consistent with findings from other studies [33,34]. The decrease in gloss is attributed to the flattening of the pits and the reduction in reflectivity caused by heat treatment [35].

The color change of wood is the most intuitive feature in the weathering process. After 12 h of weathering, the surface of the wood shows an obvious color change, and the degree of color change in the early stage is higher than in the later stage, as shown in the photograph Figure 1. Specifically, this is manifested as:

(1) The surface color of both groups of samples gradually deepened, and the color reached its deepest at 372 h, showing dark brown.

(2) The difference in surface color between the two groups of samples gradually decreased, and the difference between the two groups was the smallest at 372 h.

(3) Compared with UW, TW showed a slower color change and a smaller discoloration amplitude.

Before weathering, the lightness (L*) of TW was lower than that of UW, while the redness (a*) and yellowness (b*) were higher. During the weathering process, although both groups of samples showed similar trends in the changes in the color parameters L*, a*, b*, and ΔE, the amplitudes of color change were different between the two groups (Figure 1a,b). Specifically, the L* values of both groups decreased continuously with increasing weathering time (Figure 1a), indicating that the wood surface gradually darkened. Notably, in the early stages of weathering, the decrease in L* for TW was relatively smaller compared to UW, suggesting that heat treatment effectively delayed the darkening of wood in the short term. Although the lightness loss of TW (22.59%) was smaller than that of UW (34.18%) after 372 h of weathering, their L* values converged during the 324–372 h weathering period. This indicated that heat treatment had a limited effect on mitigating the decrease in surface lightness during long-term weathering.

In addition to changes in lightness, yellowing of the wood surface was also observed in this study. The b* values of both groups initially increased, then gradually decreased and stabilized. Throughout the entire weathering period, TW consistently displayed lower b* values than UW, and after 372 h of weathering, UW had undergone a yellowing rate of 119.85%, which was approximately twice that of TW (58.93%). This indicates that heat treatment had a sustained effect in inhibiting yellowing, consistent with previous research findings [24].

By comparison, the changes in a* values exhibited more complex, stage-specific characteristics: a rapid increase in the first 12 h, a significant drop between 12 and 24 h, followed by a gradual increase with extended weathering time. Although the changes in a* values were fluctuating, the overall trend was still toward reddening. It was noteworthy that, although the absolute a* values of TW were generally higher than those of UW, the reddening rate of TW remained consistently lower. After 372 h of weathering, the reddening rate of TW (132.35%) was markedly lower than that of UW (208.48%), which suggests that the heat treatment had the effect of slowing down the reddening of wood in weathering.

Furthermore, as shown in Figure 1b, the ΔE values of both groups increased with weathering time, with the rate of increase gradually decreasing, which is consistent with expectations. The total color change was relatively smaller for TW (ΔE = 24.05) compared to UW (ΔE = 37.78), further confirming that heat treatment mitigated color changes in wood. This is also consistent with the mechanism of lignin degradation and carbonyl formation proposed by Pandey et al. [36]. It is worth noting that lighter-colored wood is more susceptible to changes in surface color and lightness [37]. During heat treatment, the degradation of wood fibers enhances light scattering, resulting in a significant decrease in gloss. Additionally, the gloss of UW, which was initially higher, gradually decreased with increasing weathering time. In contrast, the gloss of TW initially increased and then slowly decreased, but overall remained higher than that of UW, as shown in Figure 1c. This difference is consistent with previous studies [38,39]. For example, Kucuktuvek et al. pointed out that, except for 230 °C, other heat treatments can enhance the gloss of spruce during weathering. After 372 h of weathering, the gloss of TW increased by 17%, whereas that of UW declined by 8.85%. During weathering, the erosion of the heat treatment-formed rough surface and the removal of loose degradation products may increase gloss by exposing a smoother underlying layer. Conversely, the decrease in gloss is mainly caused by light scattering from wood surface abrasion and erosion. Overall, heat treatment can significantly improve the color stability and gloss of wood.

### 3.2. Characterization of Water-Soluble Extracts by UV-Vis

**Figure 2:**
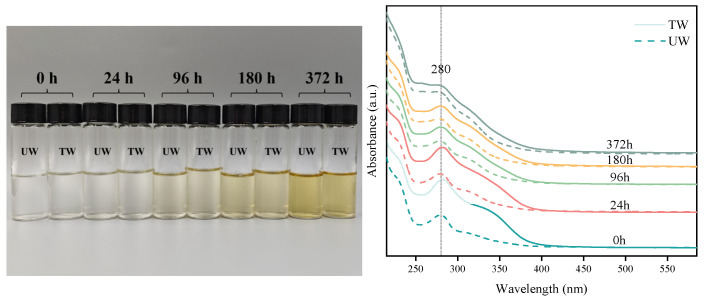
The color and UV-Vis absorption spectra of water-soluble extracts from samples at different weathering times. UW is represented by – –; TW is represented by —.

Extractives, as a key factor affecting the discoloration behavior of wood, can significantly influence the uniformity of wood surface color when leached out under the action of water. Analysis of the changes in the water-soluble extracts revealed that before weathered, the extract solution of TW was light yellow, while that of UW was transparent and colorless, as shown in Figure 2—0 h. This difference is mainly due to the degradation of the original extractives and hemicellulose, as well as the condensation of lignin during the heat treatment process. These increase the concentration of chromophores and auxochromes, such as chromogenic substances containing carbonyl, carboxyl, unsaturated double bonds, and aromatic rings [40]. UV spectroscopic analysis reveals that both sets of extracts exhibit a strong absorption peak near 280 nm, suggesting the presence of phenolic structures [41]. Additionally, the absorption intensity of TW at this wavelength is significantly higher than that of UW. This indicates that, at this temperature, heat treatment causes the wood to contain more chromophoric substances, which have a stronger UV absorption capacity at 280 nm. This also aligns with previous reports that heat treatment significantly affects the antioxidant activity of extracts [42]. It is worth noting that heat treatment not only causes the absorption peak at 280 nm to shift towards longer wavelengths (to 282 nm) but also induces the emergence of a new shoulder peak in the 330–350 nm region. The appearance of this shoulder peak corresponds to changes in phenolic aldehydes (such as pinoresinol), unsaturated ketones, quinones, stilbenes, and other conjugated carbonyl structures [43].

As the weathering time increased, the color of the extract solution from both groups of samples gradually darkened and showed a tendency to yellowing, reaching the highest degree of yellowing at 372 h. This phenomenon is not only related to the degradation and oxidation of the chemical components of wood [44,45], but is also affected by the continuous accumulation of its degradation products. However, interestingly, the absorption intensity at 280 nm decreased with increasing weathering time, and the absorption intensity of TW was always greater than that of UW at this wavelength. Meanwhile, the absorption peak continuously shifted towards shorter wavelengths during weathering (280 nm → 278 nm → 277 nm → 273 nm), indicating that the chromophoric substances formed during weathering have weaker UV absorption capacity. Moreover, although there was no significant difference in the color of the extract solution between the two groups in the early stages of weathering, starting from 96 h, the degree of yellowing of the extract solution from TW was significantly lower than that of UW. This result not only indicates that heat treatment can delay the yellowing of the extracts but also further confirms the protective effect of heat treatment on the color stability of wood.

### 3.3. Surface Chemical Changes by ATR-FTIR

**Figure 3:**
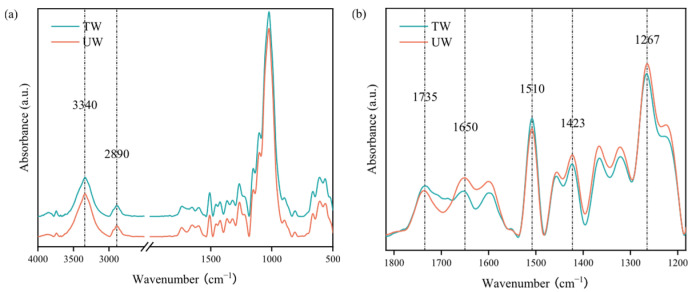
Infrared spectra of control and heat treatments, (**a**) 4000–500 cm^−1^, (**b**) 1800–1200 cm^−1^.

The changes in wood color and gloss are essentially an intuitive reflection of the evolution of its chemical components. During the heat treatment process, the evaporation of moisture and the dehydration reaction between hydroxyl groups (-OH) in hemicellulose and cellulose lead to a reduction in the number of -OH groups in the wood. This change is manifested in the infrared spectra as a weakening of the characteristic peak near 3340 cm^−1^ (Figure 3a), which is consistent with the results of Adebawo et al. [46], Barroco et al. [47], and Missio et al. [48].

The reduction in hydroxyl group content enhances the hydrophobicity of wood and significantly improves its dimensional stability and biological durability. Heat treatment not only leads to changes in hydroxyl content but also triggers the degradation of extractives and the cleavage of glycosidic bonds in hemicellulose, generating carbonyl (C=O), carboxyl (–COOH), and acetyl (–COCH_3_) groups [49], as well as acetaldehyde and furfural (–CHO) [50]. These changes were manifested as an enhancement of the characteristic peak near 1730 cm^−1^ [51,52] (Figure 3b), which is also an important contributor to the darkening of color. Xu et al. [53] pointed out that the volatile organic compounds (VOCs) generated during the heat treatment process mainly consist of acids, aldehydes, ketones, phenols, furans, alcohols, sugars, and esters, with acids being the predominant compounds. The newly formed carboxylic acids not only increase the acidity of wood but also affect polysaccharides, as evidenced by the significant weakening of the characteristic peak near 1650 cm^−1^, which corresponds to the vibration of C–O and conjugated C=O groups. The weakening of this peak is mainly due to the depolymerization of polysaccharides caused by acetic acid generated from the deacetylation of hemicellulose. The further confirmation of acetyl group removal was indicated by the weakening of the characteristic peak at 1267 cm^−1^. After heat treatment, the characteristic peak near 1510 cm^−1^ (corresponding to the vibration of the aromatic C=C in the lignin framework) slightly increased in intensity. This change is associated with the condensation of lignin (cleavage of β-O-4 bonds and demethoxylation) [54], which also influences color variation. Additionally, the degradation of hemicellulose may also indirectly contribute to the change in this characteristic peak.

During heat treatment, the pyrolysis of hemicellulose, the degradation and oxidation of extractives (such as the oxidation of phenolic hydroxyl groups to carbonyl groups, or the formation of quinone structures from adjacent hydroxyl groups [55]), and the condensation of lignin can all lead to changes in the color of the wood. This is consistent with the conclusions of Robert Nemeth et al. [56].

**Figure 4:**
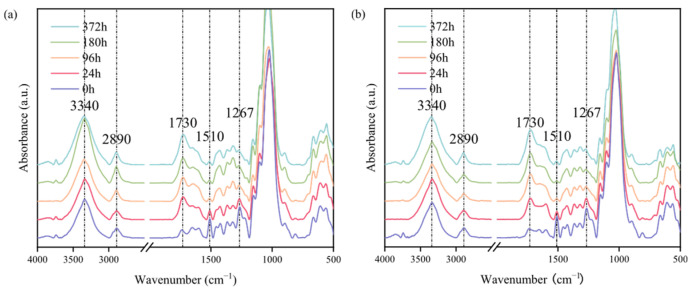
Infrared spectra of weathering at 0, 24, 96, 180, 372 h, (**a**) UW, (**b**) TW.

As can be seen from Figure 4, the chemical changes in both groups of samples during the weathering process were similar. During weathering, among the three major components of wood, lignin degraded first and to the greatest extent (especially in the early stages of weathering). The characteristic peak near 1510 cm^−1^, which is related to the vibration of the aromatic framework C=C in the lignin, generally showed a downward trend. Specifically, after 24 h of weathering, this peak dropped significantly, a phenomenon mainly attributed to the photodegradation of lignin.

The degradation of lignin significantly affected the changes in the characteristic peak near 1730 cm^−1^ (C=O of non-conjugated ketones, aliphatic carbonyl groups, and ester groups) [24], and the peak intensity in TW was lower than that in UW. This is consistent with the observation that the total color change (ΔE) of TW is smaller than that of UW after 24 h of weathering. During this period, the extractives also played a role in retarding color change (the absorption peak intensity at 280 nm in the UV spectrum decreased slightly). As weathering proceeded, the peaks at 1510 cm^−1^ and 1730 cm^−1^ did not change significantly and only slightly enhanced after 372 h of weathering. The main reason reflected in this process is that the decrease in lignin content on the wood surface after 24 h of weathering led to a slowdown in the rate of degradation as well as in the rate of carbonyl production. As weathering time progressed, the degradation of hemicellulose and cellulose exposed on the wood surface led to the formation of aldehyde and ketone groups on the C2 and C3 carbon atoms of the pyran or furan units, and these carbonyl compounds contributed to the increased absorbance at 1730 cm^−1^ [57]. The degradation of hemicellulose and cellulose also increased the relative lignin content. This explanation is supported by the continuous weakening trend of the peak at 1267 cm^−1^ (C–O vibration in the aromatic ring of lignin and hemicellulose) and the increase in intensity of the peak at 1510 cm^−1^ (C=C in the lignin aromatic framework) after 372 h of weathering. In addition, the changes at 1730 cm^−1^ were also contributed to by extractives [58]. Throughout the weathering process, the formation of the carbonyl band confirms the photo-oxidative action on the wood surface. An important finding was that after 372 h of weathering, the characteristic peak near 1730 cm^−1^ in TW was significantly higher than that in UW, which may be due to the higher initial peak intensity of TW.

Further analysis revealed that the changes at 3340 cm^−1^ (hydroxyl groups in cellulose and hemicellulose) and 2890 cm^−1^ (methyl and methylene groups in cellulose and hemicellulose) in the early stages of weathering were not significant. This further confirmed the conclusion that ‘lignin is the main component degraded during the weathering process’. It is worth noting that after 180 h of weathering, the characteristic peak near 3340 cm^−1^ in UW increased (Figure 4a), which may be due to the formation of surface cracks that allowed moisture to penetrate. In contrast, TW maintained relatively stable chemical properties due to its lower hemicellulose content.

Analysis of the infrared spectra indicated that the photo-degradation behavior of heat-treated wood during weathering was similar to that of untreated wood (natural wood). In addition, the thermal degradation of hemicellulose during the heat treatment process promoted the photo-degradation of lignin on the wood surface during the early stages of weathering. The formation of carbonyl groups was mainly associated with the degradation of lignin, and changes in hemicellulose and extractives also had a certain impact.

The wood cell wall is mainly composed of cellulose, hemicellulose, and lignin, with cellulose accounting for approximately 50% of the cell wall components. The crystallinity of cellulose is an important crystalline structural parameter that not only significantly influences the properties of wood [59,60,61] but also its resistance to environmental factors. The transcrystallinity index (TCI) and lateral order index (LOI), measured from infrared spectra, are shown in Table 2. The degradation of hemicellulose and the recrystallization of the amorphous fraction of cellulose during heat treatment resulted in a small increase in TCI and LOI. The lack of crystallinity is an important reason why the thermal stability of hemicellulose is lower than that of cellulose, and the increase in crystallinity contributes to the dimensional stability of wood. The TCI and LOI of both groups of samples showed a tendency to first increase and then decrease with the increase in weathering time. The increase in TCI and LOI was mainly related to the increase in the relative content of cellulose, whereas their decrease might originate from the increase in amorphous cellulose content, which might lead to the enhancement of hydrophilicity of the wood surface. It is noteworthy that the TCI of TW was relatively stable during the weathering process. This stability stems from the pre-degradation of heat-labile components, which reduced variation in relative crystallinity and consequently mitigated chemical degradation.

### 3.4. Crystallinity Characterization by XRD

**Figure 5:**
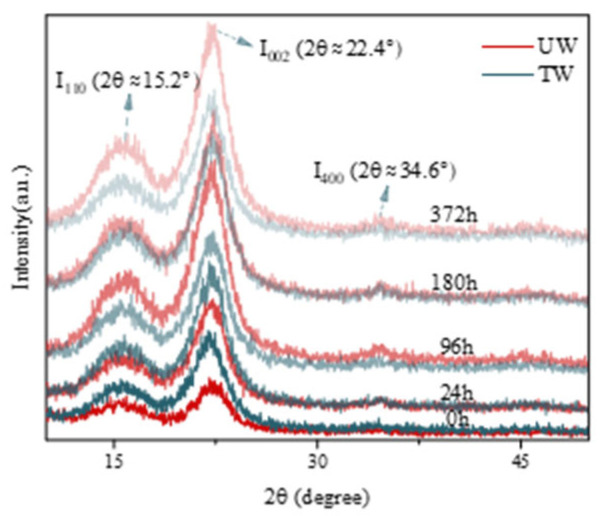
T X-ray diffractograms of UW and TW at different weathering times.

The characteristic diffraction peaks near 15.2° (large broad peak), 22.4° (large sharp peak), and 34.6° (small sharp peak) of both groups of samples can be clearly observed from Figure 5. After heat treatment, the position of the (200) diffraction peak on the crystal face of cedar cellulose was not shifted, indicating that the crystal structure was not damaged. However, the intensity of the (200) diffraction peak increased slightly after heat treatment, indicating that TW had a higher degree of crystallinity than UW [30]. With the increase in weathering time, the (200) diffraction peak showed an increasing and then decreasing trend, which corroborated the changes in TCI in Table 2.

### 3.5. Structural Analysis by SEM

**Figure 6:**
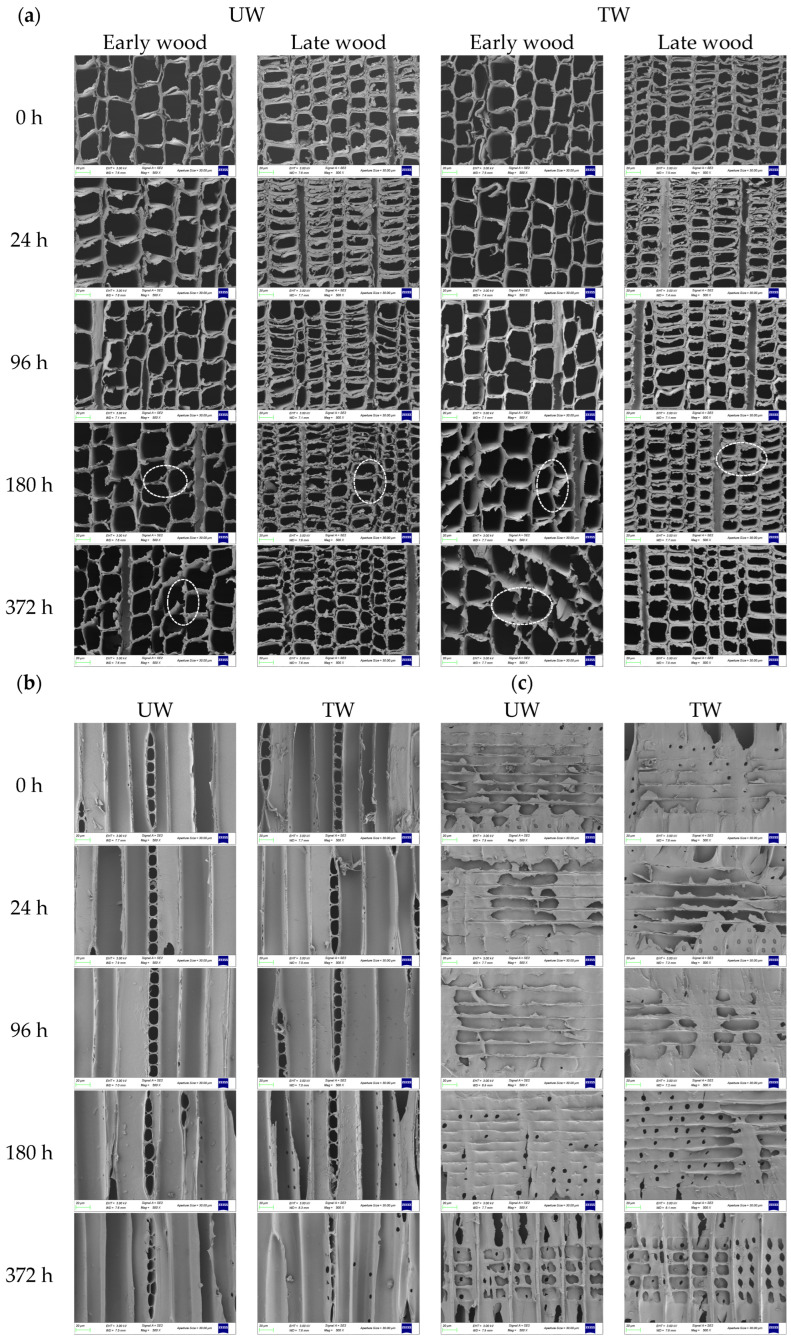
Surface morphology at 500× Magnification for UW and TW at different weathering times, (**a**) cross section; (**b**) tangential section; (**c**) radial section.

The surface morphology of the sample is shown in Figure 6. It can be clearly seen that the surface of the fir becomes rougher after heat treatment, and this roughening originates from the thermoplastic behavior of lignin [62]. On the whole, in the early stages of weathering, there was no significant change in the surface morphology of both groups of samples, but they then showed obvious deterioration after 96 h. The phenomena of thinning of cell walls, separation of true middle lamella, rupture of pits, and deformation of cells became more and more obvious with the prolongation of weathering time.

Upon heat treatment, the cell walls of earlywood tracheids in the fir cross section were slightly thinned (Figure 6a—0 h), which may be due to hemicellulose degradation. Compared with earlywood, latewood tracheids showed no significant changes, which was related to their thicker cell walls, smaller lumen diameters, and more compact structure. Kurata et al. [63] showed that the content of the three major components in the cell walls of earlywood and latewood (Cryptomeria japonica) were basically the same, but the cellulose content in the latewood was slightly higher than that in earlywood (48.3% vs. 45.0%), and lignin content was slightly lower in earlywood [64,65]. More importantly, more hemicellulose was formed through strong hydrogen bonding and electrostatic interactions in the cell walls of the latewood than in earlywood, which made latewood more thermally stable. After 96 h of weathering, the cell walls continued to thin, but the cell structure remained intact. The degree of separation of true middle lamella increased with weathering time (Figure 6a—180 h), where the separation of true middle lamella was mainly due to the higher lignin content in true middle lamella. The most severe cell wall deterioration was observed after 372 h of weathering. However, it should be noted that the complete structure of the earlywood tracheid was more severely damaged in TW compared to UW.

On the tangential section, in addition to the thinning of ray parenchyma walls (Figure 6b), longitudinal cracks in the pits and the enlargement of cell corners were also observed. The longitudinal cracks were mainly due to the relatively thinner cell walls in the pit regions and the fact that the cracks propagated along the direction of the microfibrils, while the enlargement of the cell corners was related to their higher lignin content [66,67,68]. After 372 h of weathering, the integrity of the cell wall structure was significantly disrupted, with obvious ruptures and severe cell deformation. It is worth noting that compared with UW, the cell wall deformation in TW seemed more severe after 372 h of weathering. This may be due to the degradation of hemicellulose during the heat treatment process, which weakened the ability to support the cell wall structure.

The above degradation phenomena were also present in the radial section, and the degradation in the radial section was much more severe than that in the tangential section (Figure 6c). After 96 h of weathering, cracks appeared on the tracheids of UW, while no such cracks were observed in TW, which further confirmed the protective effect of heat treatment on the wood in the early stages of weathering. After 180 h of weathering, more cracks in the pits of ray parenchyma walls were observed in UW, while TW had relatively fewer. By 372 h, the degradation of ray parenchyma walls was significantly higher than that of tracheids, mainly because ray parenchyma walls had only a very thin primary cell wall and no secondary cell wall [69].

### 3.6. Mechanisms of Degradation

During artificial weathering, the degradation behavior of wood and heat-treated wood is similar, with the degradation of lignin being the most significant. The phenolic conjugated structure in lignin absorbs UV and generates phenoxy radicals. These radicals can not only induce the cleavage of C–C and C–O bonds in lignin molecules but also be converted into quinone-type structures in the presence of oxygen. In addition, compared with cellulose, hemicellulose is more susceptible to photodegradation. This degradation intensifies water infiltration, driving the migration and diffusion of extractives to the surface.

The increase in condensed lignin structure and unsaturated phenolic substances during the heat treatment process may explain the significantly smaller color change in heat-treated Chinese fir during weathering. On the one hand, the increase in C–C bonds in condensed lignin can reduce the oxidation of side chains [70,71], thereby reducing the generation of active intermediates that cause yellowing. On the other hand, the polyphenolic structure in condensed lignin can quench radicals, inhibit oxidative chain reactions, and thus reduce the formation of quinone chromophores (i.e., block yellowing). However, the protective ability of heat treatment in long-term weathering is insufficient, which is related to the degradation of hemicellulose and part of cellulose during vacuum heat treatment, leading to a weakened ability to maintain cell structure. At the same time, the continuous degradation of lignin and the leaching of extractives during weathering further aggravate the cell structure damage of heat-treated wood.

## 4. Conclusions

The changes in the apparent properties, chemical composition, and microstructure of heat-treated wood during artificial weathering were evaluated. During heat treatment, the pyrolysis of hemicelluloses, the alteration of lignin, and the increase in carbonyls, furfural, and acetic acid collectively led to darkening, reddening, yellowing, and reduced surface gloss. During artificial weathering, untreated wood and heat-treated wood showed similar trends in surface color changes, characterized by darkening, yellowing, and reddening. However, the degree of color change in heat-treated wood was lower than that of untreated wood, while the degree of lignin degradation of heat-treated wood was higher than that of untreated wood, especially in the short-term weathering stage (particularly after 24 h of weathering). This apparent contradiction is related to the condensed lignin structure and unsaturated phenolic substances formed by heat treatment, which also indicates that heat treatment can mitigate the discoloration of Chinese fir during weathering. In addition, during the weathering process, heat treatment had the most significant inhibitory effect on yellowing and also maintained higher surface gloss of the wood. However, in long-term weathering, the protective effect of heat treatment on the appearance of Chinese fir weakened, and more importantly, it even accelerated the structural damage of the wood. In conclusion, although heat treatment can stabilize the appearance of wood in the short term, it cannot resist the structural degradation caused by long-term weathering.

This study reveals the time-dependent characteristics of the protective mechanisms of heat-treated wood during weathering—remarkable initial protective efficacy that gradually diminishes over time. However, it has certain limitations, such as the artificial weathering conditions that limit the universality of the results. To achieve long-term wood protection, it is necessary to combine heat treatment with other surface techniques. Among various methods, surface coating offers distinct advantages due to its convenient application, high efficiency, and broad applicability. However, several critical issues must be addressed when applying coatings to heat-treated wood: first, coating uniformity and adhesion, as alterations in the wood surface can readily lead to interfacial failures such as peeling or delamination; second, the chemical compatibility between the coating and the substrate, which is essential to avoid adverse reactions that might damage the wood’s interfacial structure; and third, most critically, it is imperative to address both the environmental and health impacts of the coating materials themselves and to systematically evaluate their long-term release behavior and overall lifecycle ecological impacts on thermally modified substrates. Future work will focus on exploring the synergistic effects of heat treatment combined with nano-engineering technologies, especially leveraging the unique advantages of nanoparticle modification, to develop an efficient, durable, and eco-friendly integrated protection system that meets the demands of diverse application scenarios, thereby extending both the service life and the added value of wood products.

## Figures and Tables

**Table 1:** Gloss, L*, a*, b* before and after heat treatment.

	L*	a*	b*	ΔE	Gloss
Before	81.236 (0.311)	4.878 (0.134)	19.555 (0.406)	-	4.145 (0.286)
After	68.575 (1.114)	8.215 (0.257)	25.152 (0.478)	14.272 (0.803)	3.52 (0.352)

Standard deviation in ( ).

**Table 2:** Transcrystallinity index (TCI = A1373/A2900) and lateral order index (LOI = A1423/A896) for UW and TW at different weathering times.

Weathering Time	TCI		LOI	
	UW	TW	UW	TW
0 h	0.994 (0.001)	0.996 (0.001)	1.013 (0.003)	1.014 (0.004)
24 h	0.997 (0.003)	0.999 (0.003)	1.040 (0.017)	1.039 (0.010)
96 h	1.003 (0.003)	0.999 (0.002)	1.043 (0.010)	1.050 (0.006)
180 h	1.006 (0.006)	1.000 (0.003)	1.047 (0.005)	1.041 (0.009)
372 h	1.000 (0.004)	0.999 (0.005)	1.043 (0.010)	1.038 (0.013)

Standard deviation in ( ). The absorption peak at 898 cm^−1^ is associated with the CH deformation in amorphous cellulose; the absorption peak at 1373 cm^−1^ is related to the CH deformation in hemicellulose and cellulose; the absorption peak at 1423 cm^−1^ is attributed to the vibration of the aromatic framework and the C-H in-plane deformation in crystalline cellulose.

## Data Availability

The original contributions presented in this study are included in the article. Further inquiries can be directed to the corresponding authors.
